# Functional Approaches to Discover New Compounds via Enzymatic Modification: Predicted Data Mining Approach and Biotransformation-Guided Purification

**DOI:** 10.3390/molecules30102228

**Published:** 2025-05-20

**Authors:** Te-Sheng Chang

**Affiliations:** Department of Biological Sciences and Technology, National University of Tainan, Tainan 70005, Taiwan; mozyme2001@gmail.com; Tel./Fax: +886-6-2602137

**Keywords:** biotransformation, enzymatic synthesis, glycosylation, glycosyltransferase, glycoside hydrolase, flavonoids, predicted data mining, phenolic compounds, triterpenoids

## Abstract

In the field of biotechnology, natural compounds isolated from medicinal plants are highly valued; however, their discovery, purification, biofunctional characterization, and biochemical validation have historically involved time-consuming and laborious processes. Two innovative approaches have emerged to more efficiently discover new bioactive substances: the predicted data mining approach (PDMA) and biotransformation-guided purification (BGP). The PDMA is a computational method that predicts biotransformation potential, identifying potential substrates for specific enzymes from numerous candidate compounds to generate new compounds. BGP combines enzymatic biotransformation with traditional purification techniques to directly identify and isolate biotransformed products from crude extract fractions. This review examines recent research employing BGP or the PDMA for novel compound discovery. This research demonstrates that both approaches effectively allow for the discovery of novel bioactive molecules from natural sources, the enhancement of the bioactivity and solubility of existing compounds, and the development of alternatives to traditional methods. These findings highlight the potential of integrating traditional medicinal knowledge with modern enzymatic and computational tools to advance drug discovery and development.

## 1. Introduction

Natural products have long been fundamental resources for the development of health foods and drugs. The majority of natural products are secondary metabolites found in plants [1], including alkaloids, terpenoids, and phenolic compounds [2,3]. The increasing need for new drugs to treat various human diseases has sparked a strong and sustained interest within the scientific community regarding the discovery of new bioactive compounds [4].

Isolating new compounds is the first step in the development of new drugs from plants. Traditionally, there are two strategies to isolate new compounds: isolating each natural compound from a crude extract or chemical synthesis in a laboratory [4]. The discovery and isolation of new compounds from natural resources, such as medicinal plants, is generally laborious and time-consuming. Moreover, numerous natural products have been isolated and identified over the past few decades; as a result, it has become increasingly difficult to isolate new compounds. On the other hand, organic synthesis is a promising method for the discovery of candidate compounds. However, the synthetic procedures are usually harmful to the environment; for example, due to the use of toxic chemical reagents under high pressure or high temperature. It may also be difficult to achieve high production yields for compounds with complex structures. Thus, the development of environmentally friendly approaches to obtain new compounds has become increasingly important. In contrast to empirical screening, the enzymatic biotransformation and separation of candidate targets enables more efficient approaches for the generation of novel compounds [2,3,5,6].

The structural modification of natural products using biotransformation is one effective way to produce regioselective and/or stereoselective compounds with specific bioactivities [2,3]. Hydroxylation, *O*-methylation, and *O*-glycosylation are three major modifications which occur in nature [1,7]. Various enzymes have been employed to modify functional groups (e.g., hydroxyl, glycosyl, or methyl groups) on compound backbones [2,3,5]. These enzymatic modifications often yield derivatives with significantly enhanced bioactivities or physicochemical properties, compared to the original precursors.

**Figure 1:**
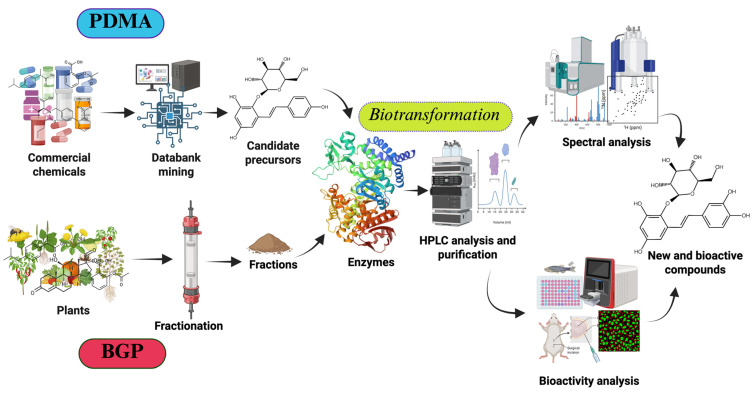
Two novel functional approaches—the PDMA and BGP—for the discovery of new and/or bioactive compounds from either commercial chemicals (the PDMA) or plant extracts (BGP).

Although enzymatic synthesis offers a good choice for the discovery of new compounds in the field of drug development, there are still some drawbacks which limit its applications. For example, there may be uncertainty regarding the biotransformation product, which may not be a new compound after the identification of its chemical structure. Therefore, a systematic method to predict the novelty of the biotransformation products before designing experiments is required. Another limitation is the high cost of some commercial pure precursors, meaning that a lower amount of raw materials can be obtained, which may also be difficult to isolate; in such cases, it is generally inconvenient to study the associated biotransformation products. Furthermore, in reality, many identified compounds are not commercially available; thus, it is much more difficult to obtain them for further applications. To overcome these limitations, the predicted data mining approach (PDMA) was recently developed as an in silico analysis technique to predict putative enzymatic biotransformation substrates for novel bioactive compounds (Figure 1) [8,9,10,11]. Indeed, the PDMA can quickly screen out candidate therapeutic agents derived from available precursors in natural sources. On the other hand, a particularly effective strategy that integrates enzymatic biotransformation with an upstream process is biotransformation-guided purification (BGP, Figure 1) [12,13,14,15]. In BGP, plant extracts are used as starting materials to find high-cost or non-commercially available compounds, with which there may be higher chances of finding novel biotransformable compounds. Through monitoring the formation of new-peak compounds, BGP can guide the direct identification and isolation of biotransformed products from complex mixtures, followed by enzymatic validation with desired properties or bioactivities. This review collected reported cases that used the PDMA or BGP to quickly produce new compounds from either commercial precursors (in PDMA cases) or plant extracts (in BGP cases), as shown in Table 1.

## 2. Finding New Compounds Using the Predicted Data Mining Approach

The scientific community is continuously pursuing novel compounds with biological activities. Enzyme-catalyzed biotransformation is a method that uses the catalytic power of enzymes to modify the structure of compounds. Biotransformation has garnered significant attention in the field of drug development due to its high specificity, efficiency, and ability to carry out reactions that are challenging in the context of chemical synthesis. However, conventional biotransformation largely depends on a time-consuming trial-and-error methodology. Biotransformation is generally used to produce known molecules with known bioactivities, resulting in low efficiency and high costs when attempting to find new bioactive compounds [2,3,5,6].

**Figure 2:**
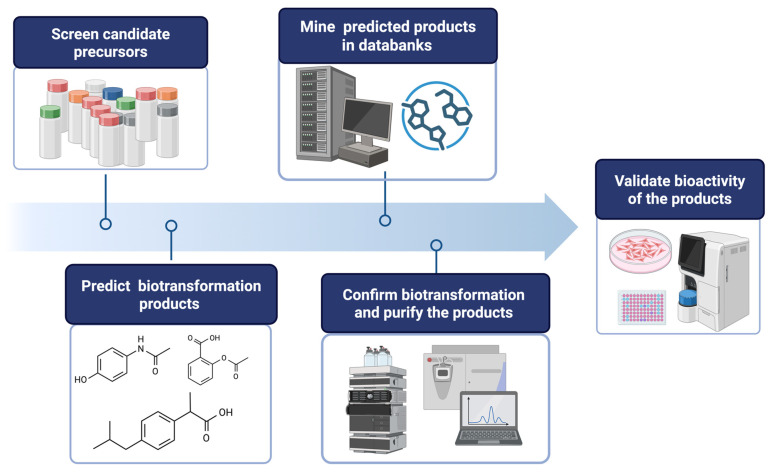
Flowchart of the predicted data mining approach (PDMA).

To overcome these limitations, the PDMA (Figure 2) has been developed. The main idea of the PDMA is to utilize the known catalytic properties of enzymes and precursor/chemical structures to predict putative products through in silico computational screening. Based on known chemical databases, this strategy aims to efficiently filter out novel compounds, which reduces the time and cost of experimental validations and prevents resource wastage.

This section delves into the core principles, general workflow, and advantages of the PDMA compared to traditional methods. Five recent studies have reported how the PDMA can be applied with respect to various enzymatic biotransformation reactions (hydroxylation, glycosylation, and methylation), demonstrating the effectiveness of the PDMA in discovering novel compounds with potential pharmacological and biotechnological applications.

The PDMA generally follows six steps (Figure 2), although the specific implementation details may vary depending on the research objectives and the enzymes used:

1. Setting Screening Criteria (selecting a suitable enzymatic reaction): Define clear screening criteria based on the target enzyme’s known catalytic mechanism, substrate preference, and desired product characteristics. These criteria may include specific functional groups, structural features, physicochemical properties of precursor compounds, and their availability at an industrial scale. For example, hydroxylation by tyrosinase (*Bm*TYR) requires precursors containing a phenyl group mimicking the structure of tyrosine; glycosylation by glycosyltransferases (GTs) requires precursors with at least one hydroxyl group that can be glycosylated; and *O-methylation* by *O*-methyltransferases (OMTs) requires precursors with a catechol structure.
2. Screening Candidate Precursors: Based on the defined screening criteria, potential candidate precursors can be screened from commercial chemical or natural product databases. These databases usually contain a vast amount of compound structures and related information. In some cases, customized catalogs of commercially available compounds are used.
3. Predicting Biotransformation Product Structure: For the selected candidate precursors, the structures of potential biotransformation products under the action of the target enzyme are determined using on-line chemical drawing software supplying by databases, such as Reaxys^®^ or SciFinder^®^. This step requires researchers to have a certain knowledge of the enzyme’s catalytic mechanism; for instance, *Bm*TYR primarily catalyzes *ortho*-hydroxylation, GTs catalyze the transfer of sugar moieties, and OMTs catalyze the transfer of methyl groups.
4. Verifying Product Novelty: The predicted biotransformation product structures are uploaded to chemical databases (e.g., Reaxys^®^, PubChem^®^, or SciFinder^®^) in order to verify their novelty, confirming whether each product is a known compound. Only precursors that yield novel derivatives are further selected for subsequent experimental validation.
5. In Vitro Biotransformation and Product Identification: The selected precursors are reacted with the target enzyme in vitro. The biotransformed products are analyzed using isolation methods, such as high-performance liquid chromatography (HPLC). Once the putative new compounds are purified, their chemical structures can be identified using techniques such as mass spectrometry (MS) and nuclear magnetic resonance (NMR).
6. Bioactivity Evaluation: Alternatively, the identified compounds may undergo bioactivity testing to evaluate their potential application value. The tested activities may include antioxidant, anti-inflammatory, anticancer, and anti-diabetic properties, among others.

**Figure 3:**
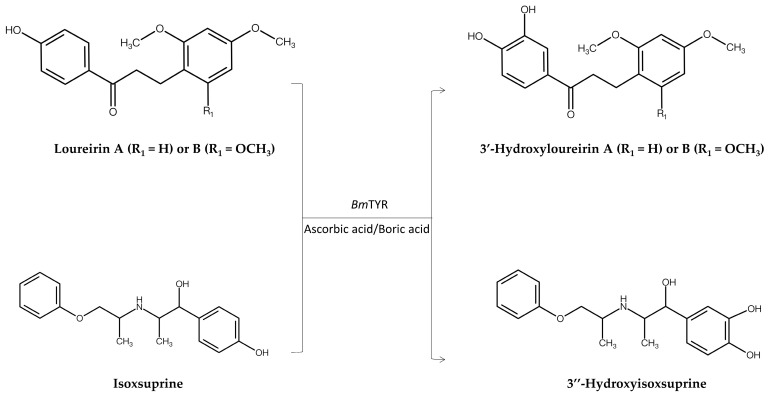
Enzymatic synthesis of new catecholic compounds from loureirin A/B or isoxsuprine by *Bm*TYR, determined through the PDMA [8,9].

The first reported case study using the PDMA involved the hydroxylation of Dragon’s Blood components by *Bm*TYR to enhance their antioxidant and anti-α-glucosidase activities (Figure 3) [8]. Kim et al. have developed an efficient hydroxylation system for flavonoids using *Bm*TYR in the presence of ascorbic acid and boric acid [17]. Generally, *Bm*TYR catalyzes the transformation of monophenolic compounds to catecholic compounds, which are quickly converted to quinone products by the same enzyme. As such, it is difficult to obtain the unstable intermediate catecholic compounds. However, in the presence of both ascorbic acid and boric acid, the oxidized quinone products would be reduced back to the catecholic structure, which is chelated by boric acid. Therefore, in the presence of both ascorbic acid and boric acid, *Bm*TYR catalyzes the transformation from monophenolic compounds to catecholic compounds as stable intermediate products. The study revealed that *Bm*TYR is a promiscuous biocatalyst for flavonoid hydroxylation. Thus, *Bm*TYR was selected for use in the first trial to validate the main concept of the PDMA. The catalytic criteria of *Bm*TYR included its containing a phenyl group mimicking tyrosine. The PDMA further screened 475 compounds and identified loureirin A and loureirin B as candidate precursors. Data mining further confirmed the predicted products, 3′-hydroxyloureirin A and 3′-hydroxyloureirin B, as novel flavonoids. Thus, biotransformation was conducted, and the experimental validation confirmed that *Bm*TYR indeed converts loureirin A and loureirin B into these two flavonoids. In addition, bioactivity assays revealed that these new compounds exhibited significantly higher antioxidant activity than ascorbic acid and possessed remarkable anti-α-glucosidase activity. Overall, the PDMA allowed for rapid screening of hundreds of compounds to identify two potential novel flavonoids obtained via enzymatic biotransformation.

Therefore, the PDMA was further applied to facilitate *ortho*-hydroxylation by *Bm*TYR to generate compounds with enhanced antioxidant and anti-inflammatory activities (Figure 3) [9]. This study screened 764 commercially available chemical compounds that are suitable as substrates of *Bm*TYR in order to produce new catecholic compounds. Only isoxsuprine was selected as a potential substrate, due to its phenolic structure. The PDMA predicted that *Bm*TYR would catalyze the hydroxylation of isoxsuprine, yielding a novel catechol product. Experimental validation confirmed that isoxsuprine could be biotransformed into novel compound 3″-hydroxyisoxsuprine. Bioactivity assays further demonstrated that 3″-hydroxyisoxsuprine exhibited 40-fold higher antioxidant activity and 10-fold higher anti-inflammatory activity than isoxsuprine.

**Figure 4:**
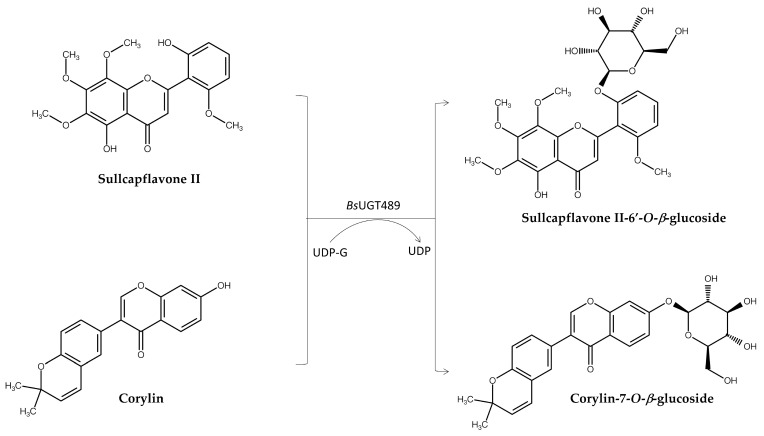
Enzymatic synthesis of new glucosides from corylin or skullcapflavone II by *Bs*UGT489, determined through the PDMA. UDP-G: uridine diphosphate glucose. UDP: uridine diphosphate.

The PDMA was further applied to screen 412 commercial natural compounds for potential glycosylation by a bacterial glycosyltransferase (GT), *Bs*UGT489, from the *Bacillus subtilis* American Type Culture Collection (ATCC) 6633 strain. In nature, many compounds can be glycosylated by GTs to form glycosides, and the glycosylation of molecules is considered a promising strategy to obtain glycosylated derivatives with greatly improved water solubility [18]. Thus, the glycosylation of medicinal compounds may also improve their oral bioavailability [19]. Based on the advantages described above, glycosylation coupled with the PDMA to find new and bioactive glycosides has also been performed. When coupling the abovementioned GT with the PDMA, the screening criteria included compounds with industrial-scale availability, possessing one hydroxyl group, and the predicted glucoside product being novel in the SciFinder^®^ database. Corylin was predicted to be glycosylated by *Bs*UGT489, thus yielding a novel glucoside. Molecular docking analysis also predicted corylin as a compatible substrate. Experimental validation and structural analysis further validated the new glucoside, corylin-7-*O*-*β*-glucoside (Figure 4) [10]. This new glucoside demonstrated significantly enhanced aqueous solubility, retained anti-inflammatory activity, and exhibited more potent anti-melanoma activity than its precursor, corylin. Through a traditional approach, another clinical team also identified corylin from 22 studied compounds as a potential compound promoting longevity in eukaryotic cells [20]. The PDMA can indeed be considered as a cost-effective strategy to quickly survey potential compounds for drug development, which could be extended to clinical trials or disease therapy in the future.

Another report screened 1143 natural compounds and showed that skullcapflavone II glycosylated by *Bs*UGT489 may produce a highly soluble and anti-melanoma flavone glucoside (Figure 4) [11]. This study utilized the PDMA combined with molecular docking analysis to predict glucoside derivatives of skullcapflavone II as novel flavonoid compounds with higher binding potential to oncogenic proteins. Experimentally, *Bs*UGT489 was proven to be effective in glycosylating skullcapflavone II into a novel compound, skullcapflavone II-6′-*O*-*β*-glucoside. Moreover, the novel glucoside exhibited 272-fold higher aqueous solubility than its precursor and demonstrated noteworthy anti-melanoma activity.

**Figure 5:**
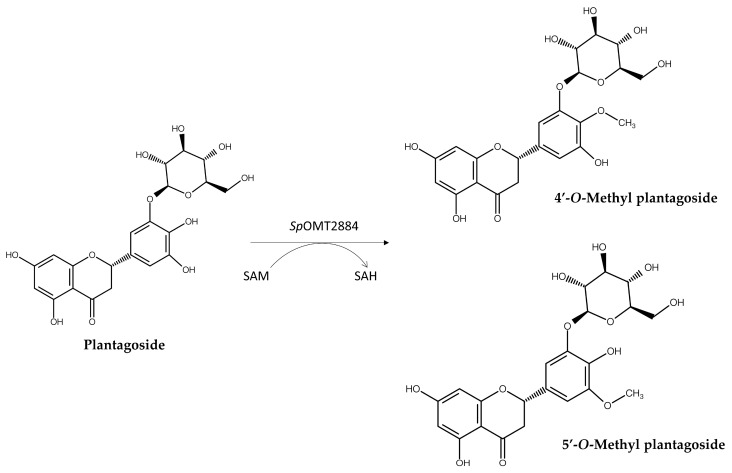
Enzymatic synthesis of new methyl compounds from plantagoside by *Sp*OMt2884, determined through the PDMA. SAM: *S*-adenosylmethionine. SAH: *S*-adenosylhomocysteine.

As the PDMA is a functionally validated method for hydroxylation and glycosylation, the PDMA coupled with a bacterial *O*-methyltransferases (OMTs) has also been investigated for the discovery of new methyl compounds. Methylation is another common modification occurring in natural products [21]. In nature, methylation is mainly catalyzed by OMTs, which transfer the methyl group of a methyl donor molecule, *S*-adenosyl methionine (SAM), to an acceptor molecule [22]. Many *O*-methylated natural products have been identified with multiple bioactivities, especially plant metabolites [1,7,23,24]. The bioactivities of some natural products can be greatly improved after *O*-methylation using bacterial OMTs [25]. Accordingly, a recent study utilized the PDMA coupled with a *Streptomyces peucetius O*-methyltransferase (*Sp*OMt2884) to screen 4364 commercially available natural compounds with catechol structures as potential substrates. The screening criteria were based on the catalytic characteristics of *Sp*OMt2884 and the novelty of the predicted methylated products in the SciFinder^®^ database. Eight precursors were predicted to form novel methyl derivatives, and seven of them (including plantagoside and protosappanin B) were successfully biotransformed by *Sp*OMT2884. Two novel compounds, 4′-*O*-methyl plantagoside and 5′-*O*-methyl plantagoside, were identified in advance by NMR as new methylated plantagoside derivatives (Figure 5). This study demonstrated that the PDMA does indeed help to find new derivatives. However, it should be noted that the assessment of the bioactivity of these methylated compounds was restricted to a quick functional screening. Nevertheless, other bioactivities of these compounds could be investigated via high-throughput drug screening assays in the future.

Compared with traditional trial-and-error methods, the successful application of the PDMA across different enzyme types (hydrolases and transferases) and chemical modifications (hydroxylation, glycosylation, and methylation) highlights its broad applicability. All these applications exhibit great potential in the fields of pharmacology and biotechnology. In short, the PDMA offers the following significant advantages:

1. High Efficiency: The PDMA enables rapid in silico screening of a large number of compounds, targeting potential precursors and thereby significantly reducing the time required to find suitable biotransformation substrates.
2. Reduced Cost: Through minimizing the number of trial-and-error experiments, the PDMA helps to lower the consumption of experimental reagents, enzymes, and human resources, as well as reducing costs associated with clinical trials.
3. Predicting Novelty: The PDMA predicts whether a given product is a novel compound before experimentation, avoiding the risk of redundant research on known compounds and increasing the likelihood of discovering new entities.
4. Knowledge-Based Guidance: The PDMA can predict outcomes based on the enzyme’s characteristics, the precursor’s structure, and the experimental design, helping researchers better understand the potential results of biotransformation reactions.
5. Applicable to Various Enzymes and Reactions: The PDMA concept is not limited to specific enzymes or reaction types. It can be adapted based on the catalytic properties of different enzymes and applied to various biotransformation processes, including hydroxylation, glycosylation, and methylation.

However, using the PDMA also presents certain challenges. Although the PDMA can predict the novelty of biotransformation products, it lacks information about the likelihood of the selected enzyme successfully catalyzing the precursor. In addition, all successful studies that employed the PDMA have utilized precursors readily available from local and commercial chemical catalogs. This reliance on standard sources has consequently limited the range of novel products discovered. Future developments of the PDMA should focus on its enhancement through integrating advanced computational simulations (e.g., molecular docking and molecular dynamics), thus enabling more accurate precursor–enzyme interaction modeling. Additionally, incorporating comprehensive chemical/biological databases as the sources of chemical precursors will improve the PDMA’s predictive efficiency.

## 3. Finding New Compounds via Biotransformation-Guided Purification

In most natural product biotransformation processes, pure compounds (precursors) are necessary substrates. There are two major ways to obtain these pure compounds: purifying them from natural sources or purchasing them from chemical companies. However, traditional methods for isolating pure compounds from plants are often laborious, time-consuming, and yield limited amounts of the desired molecules for drug discovery [1]. Moreover, few new compounds are typically identified from such purified compounds, as most have already been characterized. Meanwhile, for commercially prepared pure compounds, the high cost of some pure precursors can limit such investigations. Even when precursors are available, these compounds may not be novel after biotransformation. All of these disadvantages lead to a high risk of failure in isolating new drugs.

**Figure 6:**
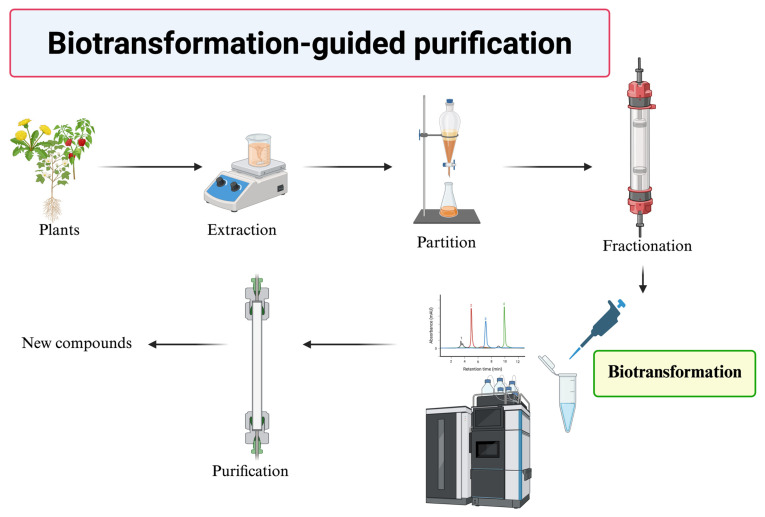
Biotransformation-guided purification (BGP) workflow.

In contrast, plant crude extracts serve as another low-cost precursor resource; however, they generally contain complex mixtures of various compounds, making them difficult to use in biotransformation. To overcome this challenge, BGP has emerged as a complementary strategy for generating novel bioactive compounds (Figure 6). BGP represents an innovative approach that integrates enzymatic biotransformation with traditional purification techniques (e.g., chromatography). The workflow of BGP involves the following: (1) crude extraction of the natural source, (2) partial purification or fractionation of the crude extract, (3) enzymatic biotransformation of the fractionated extract, and (4) purification and identification of the biotransformed products (Figure 6). In contrast, chemical purification involves multiple-step chromatography to isolate each natural compound in a step-by-step manner.

BGP allows biotransformable compounds to be quickly extracted from crude extracts. For example, the Chinese medicinal fungus Ganoderma lucidum possesses over 300 fungal triterpenoids; however, only three Ganoderma triterpenoid glycosides have been found so far. This phenomenon may be due to a lack of specific glycosyltransferases (GTs) required to form fungal terpenoid glycosides. Indeed, while more than 20,000 GT1 enzymes have been identified [26], only a few GT1 genes belong to fungi, which may explain why fewer terpenoid glycosides are found in fungi. Thus, *Ganoderma lucidum* extract is considered a suitable crude extract for use in BGP, coupled with recombinant GTs to produce new glycosides.

**Figure 7:**
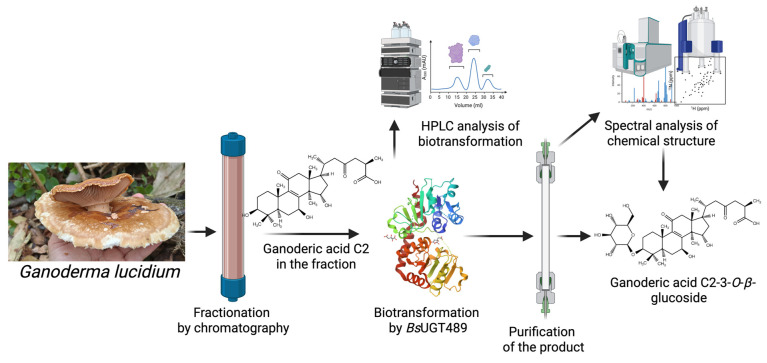
Enzymatic synthesis of a new glucoside from *Ganoderma lucidum* by *Bs*UGT489 through BGP [12].

In this line, Ding et al. (2023) [12] have successfully utilized BGP coupled with a *Bacillus* GT, *Bs*UGT489, to generate novel *Ganoderma* triterpenoid saponins from a commercial *Ganoderma* extract (Figure 7). The commercial *Ganoderma* extract was partially separated into three fractions via preparative HPLC equipped with a C18 column, and these fractions were directly biotransformed by recombinant *Bs*UGT489. One major biotransformation product could be purified from the fraction and was identified as a novel saponin, ganoderic acid C2 (GAC2)-3-*O*-*β*-glucoside, as confirmed by NMR and mass spectral analyses. Further validation revealed that commercial GAC2 indeed could be biotransformed by BsUGT489 into four novel saponins, including GAC2-26-*O*-*β*-glucoside, GAC2-15-*O*-*β*-glucoside, GAC2-3-*O*-β-glucoside, and the novel diglucoside GAC2-3,15-*O*-*β*-diglucoside. Importantly, GAC2-3-O-β-glucoside and GAC2-3,15-*O*-*β*-diglucoside exhibited significantly higher aqueous solubility (17- and 200-fold, respectively) than GAC2. Additionally, GAC2-3-*O*-*β*-glucoside retained significant anti-α-glucosidase activity, comparable to that of the anti-diabetes drug acarbose. This case study reveals that the efficient BGP not only facilitates the discovery of novel compounds but also allows for the isolation of biotransformable compounds with desirable physicochemical properties, such as improved solubility or enhanced bioactivity.

In addition to GT1s, GHs are another type of enzyme which are capable of glycosylating flavonoids [18,27]. In particular, while GT1s glycosylate flavonoids via a *β*-glycosidic linkage, GHs can glycosylate flavonoids via an *α-*glycosidic linkage. Both *α*-glycosidic and *β*-glycosidic flavonoids are more soluble than flavonoids [10,19]. Notably, GT1s use expensive uridine diphosphate-glucose (UDP-G) as the sugar donor [28], whereas GHs use cheaper sugars (e.g., starch, maltodextrin, maltose, and sucrose) as donors during glycosylation [27]. Therefore, GHs are generally used for the bio-industrial production of glycosylated molecules. Moreover, all plant glycoside derivatives of natural products can be produced by their own GT1s, which means that plant glycosides are always of *β*-form. Thus, GHs are suitable promiscuous enzymes for the production of new *α*-glycosides from natural compounds. The carbohydrate-activating enzyme (CAZY) databank records 1.9 million GHs, classified into 190 distinct families [29]. As many putative enzymes were still unverified, new glycosylated derivatives of natural products that bear suitable structures as acceptors can be designed and synthesized with novel enzymes in the future. There have been several successful cases in this context:

Amylosucrase (AS, E.C. 2.4.1.4) is a versatile sucrose-hydrolyzing enzyme that belongs to GH family 13 (GH13) [30,31,32,33,34]. AS can catalyze the synthesis of *α*-1,4-glucans using sucrose as the sole substrate. Furthermore, it can mediate the glycosylation of various small molecules with sucrose. Recombinant *Deinococcus geothermalis* amylosucrase (*Dg*AS) is a very famous AS for the glycosylation of natural products [30,34]. Some natural products have been demonstrated to be suitable substrates of *Dg*AS, including simple phenolics, such as hydroquinone [35]; flavonoids, such as catechin [36], rutin [37], daidzin [38,39,40], and isoquercitrin [33].

**Figure 8:**
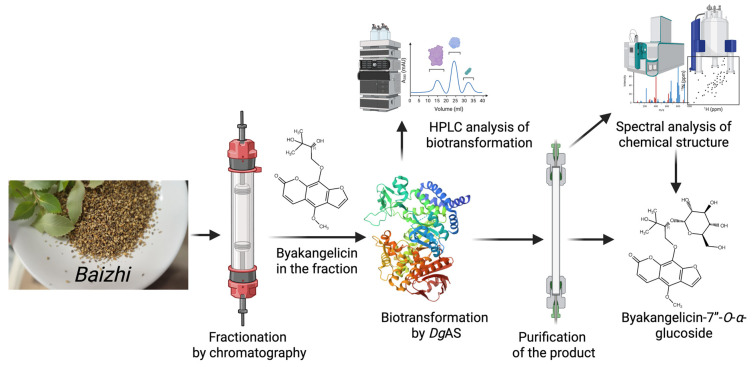
Enzymatic synthesis of a new glucoside from Baizhi herb by *Dg*AS through BGP.

Based on the powerful glycosylation activity of *Dg*AS toward natural products, Chang et al. (2024) [13] employed the BGP approach with *Dg*AS to produce a novel glycoside from extracts of the Chinese herb Baizhi (*Angelica dahurica*) (Figure 8). Baizhi is known for its anti-inflammatory and analgesic properties, and the researchers aimed to enhance the properties of its bioactive constituents through glycosylation. Initial biotransformation of the crude Baizhi extract with *Dg*AS yielded limited products, due to unknown inhibition by chemicals or compounds in the extract. Thus, the extract was further partitioned into four fractions by HPLC, and the second fraction yielded a significant amount of a putative novel compound upon reaction with *Dg*AS. The biotransformed compound was confirmed as byakangelicin-7″-*O*-*α*-glucopyranoside (BG-G3), a new *α*-glucoside derivative of byakangelicin (Figure 8). Further experiments validated that *Dg*AS indeed glycosylates pure byakangelicin to produce BG-G3. Remarkably, the aqueous solubility of byakangelicin-7″-O-α-glucoside was found to be over 29,000-fold greater than that of byakangelicin. This significant improvement in solubility indicated clinical potential for enhanced bioavailability and therapeutic efficacy. This study exemplifies how the BGP concept may help to overcome the unknown inhibition or low efficiency of enzymes within crude extract, quickly targeting novel derivatives or biotransformable compounds with improved pharmaceutical properties.

As can be seen from the above Baizhi case, the biotransformation efficiency can be improved when using fraction mixtures as precursors. This might be due to the removal of certain competitive substrates or inhibitors through chromatographic fractionation, such that the fraction mixtures became more suitable for biotransformation via BGP. BGP can indeed save a lot of effort and cost for target compounds (precursors), instead of using commercial pure compounds or isolated precursors individually.

**Figure 9:**
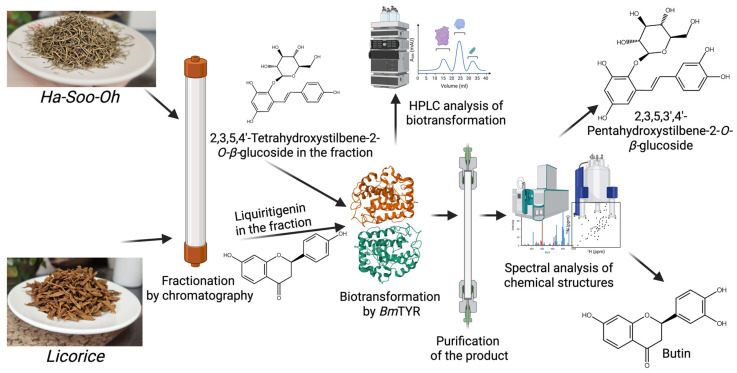
Enzymatic synthesis of new catecholic compounds from licorice or Ha-Soo-Oh by *Bm*TYR through BGP [14,15].

In addition to glycosylation, Wu et al. [15] used BGP coupled with *Bm*TYR (ortho-hydroxylation) to produce butin from licorice (*Glycyrrhiza*) (Figure 9). Licorice is a widely used traditional Chinese medicine which contains various flavonoids with valuable bioactivities [41]. Thus, the researchers employed *Bm*TYR to catalyze the biotransformation of a commercial licorice extract. However, *Bm*TYR failed to catalyze a significant product from either the methanol crude extract or the ethyl acetate extract. To overcome this, the ethyl acetate extract was further fractionated using Sephadex LH-20 chromatography. One fraction yielded a significant biotransformation product upon incubation with BmTYR. This product was subsequently purified using preparative C-18 Via high-performance liquid chromatography (HPLC) and was identified as butin (3′-hydroxyliquiritigenin) through NMR and MS analyses. The study further confirmed that butin was produced from liquiritigenin—a major flavonoid in licorice—through BmTYR-catalyzed hydroxylation (Figure 9). Notably, butin exhibited over 100-fold stronger antioxidant activity compared to its precursor, liquiritigenin. Moreover, the BGP process yielded a significantly higher amount of butin (0.15% from licorice medicine) when compared to traditional isolation methods from other natural sources (0.0001% to 0.0055%). This case study highlights that BGP can be used not only to identify novel bioactive molecules but also to enhance their bioactivity and achieve a higher yield of desired compounds at low cost.

Wu et al. (2022) [14] also applied BGP to the Chinese herb Ha-Soo-Oh (*Polygonum multiflorum*) using *Bm*TYR as a biocatalyst, leading to the discovery of a new stilbene glucoside, 2,3,5,3′,4′-pentahydroxystilbene-2-*O*-*β*-glucoside (PSG) (Figure 9). Direct biotransformation of the Ha-Soo-Oh extract with BmTYR resulted in the formation of a major product, which was purified via preparative HPLC and identified as PSG using mass spectrometry and NMR. Further investigations confirmed that PSG is a hydroxylation product of 3,5,4′-trihydroxystilbene-2-*O*-*β*-glucoside (TSG)—a main stilbene glucoside in Ha-Soo-Oh, which serves as a quality indicator for this herb (Figure 9). The new compound PSG demonstrated a 4.7-fold higher 1,1-diphenyl-2-picrylhydrazine (DPPH) free radical scavenging activity than its precursor TSG. This study further underscores the utility of BGP in discovering novel bioactive compounds from traditional Chinese medicines and quickly identifying valuable precursors.

Previous studies have highlighted the following significant advantages of the BGP strategy in natural product research:

1. Discovery of Novel Bioactive Compounds: BGP facilitates the identification of new molecules that are structurally related to known bioactive precursors but possess altered or enhanced properties.
2. Enhanced Bioactivity: Enzymatic biotransformation can modify the functional groups of precursor molecules, leading to derivatives with significantly improved bioactivity, as demonstrated by the enhanced antioxidant activities of butin and PSG.
3. Improved Physicochemical Properties: BGP can be used to generate derivatives with enhanced pharmaceutical properties, such as significantly increased aqueous solubility, as seen with the Ganoderma glucosides GAC2-3-*O*-*β*-glucoside and GAC2-3,15-*O*-*β*-diglucoside, as well as byakangelicin-7″-*O*-*α*-glucoside from Baizhi.
4. Increased Yield of Active Ingredients: By selectively biotransforming a specific precursor within a complex mixture and then purifying the valuable product, BGP can sometimes lead to higher yields of the target compound when compared to direct isolation from the natural source, as observed in the production of butin.
5. Cost-Effectiveness: BGP can utilize crude or partially purified extracts as starting materials, potentially reducing the need for extensive initial purification of precursors and leading to a more economical process.
6. Efficiency in Screening Biotransformable Compounds: BGP offers an efficient method to screen complex natural extracts for compounds that can be biotransformed by specific enzymes, rather than testing expensive pure compounds individually.

However, the application of BGP also involves certain considerations. First, the success of BGP heavily relies on the choice of an appropriate enzyme with the desired catalytic activity and substrate specificity. In the case of Baizhi, the used enzyme *Dg*AS could catalyze a unique biotransformation—namely, α-glycosylation of flavonoids—which does not occur naturally in plants. Thus, applying a unique functional enzyme with BGP is a good choice to obtain new and bioactive compounds. Second, crude extracts contain various chemicals/compounds that may inhibit the activity of the biotransformation enzyme, as observed in the initial attempts with licorice and Baizhi extracts. Thus, partial purification or suitable fractionation of the extract may need to be performed before biotransformation. Additional chromatographic fractionations might also isolate more biotransformable fractions as a trade-off. Third, BGP might require optimized biotransformation conditions with respect to different herbal extracts. Even for pure compounds, the biotransformation enzyme might have different activities under optimal pH and temperature conditions. Therefore, one may need to carefully optimize the reaction parameters, such as enzyme concentration, substrate concentration, pH, temperature, and incubation time, in order to achieve efficient biotransformation. Finally, BGP may isolate complex mixtures of products from crude extracts, but efficient purification and analytical techniques are still required for the isolation and identification of each desired compound.

## 4. Conclusions

This review highlights the significant potential of the PDMA and BGP as powerful and efficient strategies for the modification and discovery of bioactive natural products. The PDMA serves as an effective in silico method for the prediction of potential biotransformation reactions, guiding the isolation of new derivatives with desired characteristics. The successful application of the PDMA in developing promising compounds such as 3′-hydroxyloureirin A and B, skullcapflavone II-6′-*O*-*β*-glucoside, 3″-hydroxyisoxsuprine, and various methylated products has demonstrated its efficacy in generating derivatives with improved anti-melanoma, anti-inflammatory, and antioxidant activities. In the future, the PDMA may be further enhanced through the integration of more advanced computational simulation techniques, such as molecular docking and molecular dynamics simulations, in order to more accurately model precursor–enzyme interactions. Furthermore, integrating more comprehensive chemical and biological databases and developing more intelligent screening algorithms will help to improve the prediction accuracy and efficiency of the PDMA. With the continuous advancement of biotechnology and computer science, the PDMA stands out as a knowledge-based method for the next generation of bioactive molecule discovery, bringing more innovation and breakthroughs in the fields of drug development, functional foods, and cosmetics, among others.

Complementarily, BGP may help to explore unknown functional compounds or rare active ingredients retained in crude extracts. BGP allows for the direct isolation of novel compounds resulting from enzymatic transformations of fractionated plant extracts, which often exhibit enhanced properties such as antioxidant activity, solubility, and bioactivity. BGP has emerged as a cost-efficient and versatile process for the discovery and isolation of novel and/or bioactive molecules from natural raw materials. Through seamlessly integrating enzymatic biotransformation with available purification methodologies, BGP can help researchers overcome the disadvantages and limitations of the methods traditionally used in natural product research. For instance, applying BGP to licorice, Ha-Soo-Oh, *Ganoderma*, and Baizhi unequivocally demonstrated it as a cost-effective strategy for yielding novel compounds with enhanced bioactivity and improved physicochemical properties. As the repertoire of characterized biocatalytic enzymes continues to expand, BGP holds immense promise for accelerating the discovery of new drugs and functional ingredients from the vast and largely untapped resources of the natural world, particularly those found in traditional medicines.

## Figures and Tables

**Table 1:** Enzymatic synthesis of new compounds using either the PDMA or BGP.

Strategy	Enzyme	Precursor	Novel Product	Properties of the New Compounds	Reference
Predicted data mining approach (PDMA)	*Bm*TYR ^1,2^	Loureirin A Loureirin B	3′-Hydroxyloureirin A 3′-Hydroxyloureirin B	Improved both antioxidant and anti-*α*-glucosidase activity	[8]
	*Bm*TYR	Isoxsuprine	3″-Hydroxyisoxsuprine	Improved both antioxidant and anti-inflammatory activity	[9]
	*Bs*UGT489 ^1,3^	Corylin	Corylin-7-*O*-*β*-glucoside	Improved both anti-inflammatory and anti-melanoma activity	[10]
	*Bs*UGT489	Skullcapflavone II	Sullcapflavone II-6′-*O*-*β*-glucoside	Improved both solubility and anti-melanoma activity	[11]
	*Sp*OMT2884 ^1,4^	Plantagoside	4′-*O*-Methyl plantagoside 5′-*O*-Methyl plantagoside	Not mentioned	[16]
Biotransformation-guided purification (BGP)	*Bs*UGT489	*Ganoderma* extract	Ganoderic acid C2-3-*O*-*β*-glucoside	Improved solubility and maintains anti-*α*-glucosidase activity	[12]
	*Dg*AS ^1,5^	Baizhi herb	Byakangelicin-7″-*O*-*α*-glucoside	Improved solubility	[13]
	*Bm*TYR	Ha-Soo-Oh herb	2,3,5,3′,4′-Pentahydroxystilbene-2-*O-β-*glucoside	Improved antioxidant activity	[14]
	*Bm*TYR	Licorice herb	Butin	Improved antioxidant activity	[15]

^1^ Recombinant enzyme isolated from *Escherichia coli*. ^2^ Genetic source: *Bacillus megaterium*. ^3^ Genetic source: *Bacillus subtilis*. ^4^ Genetic Source: *Streptomyces peucetius*. ^5^ Genetic source: *Deinococcus geothermalis*.

## Data Availability

The data presented in this study are available on request from the corresponding author.

## References

[1] Ekiert H.M., Szopa A. (2020). Biological activities of natural products. Molecules.

[2] Muffler K., Leipold D., Scheller M.-C., Haas C., Steingroewer J., Bley T., Neuhaus H.E., Mirata M.A., Schrader J., Ulber R. (2011). Biotransformation of triterpenes. Process Biochem..

[3] Cao H., Chen X., Jassbi A.R., Xiao J. (2015). Microbial biotransformation of bioactive flavonoids. Biotechnol. Adv..

[4] Luca S.V., Macovei I., Bujor A., Miron A., Skalicka-Wozniak K., Aprotosoaie A.C., Trifan A. (2020). Bioactivity of dietary polyphenols: The role of metabolites. Crit. Rev. Food Sci. Nutr..

[5] Sultana N., Saify Z.S. (2013). Enzymatic biotransformation of terpenes as bioactive agents. J. Enzym. Inhib. Med. Chem..

[6] Andres P., Francisco R., Andres G.-G., Antonio M. (2009). Microbial transformation of triterpenoids. Mini-Rev. Org. Chem..

[7] Badshah S.L., Faisal S., Muhammad A., Poulson B.G., Emwas A.H., Jaremko M. (2021). Antiviral activities of flavonoids. Biomed. Pharmacother..

[8] Wu J.-Y., Ding H.-Y., Wang T.-Y., Cai C.-Z., Chang T.-S. (2023). Antioxidant and anti-α-glucosidase activities of biotransformable dragon’s blood via predicted data mining approach. Process Biochem..

[9] Wu C.Y., Ding H.Y., Wang T.Y., Liu C.W., Wu J.Y., Chang T.S. (2024). Development of a new isoxsuprine hydrochloride-based hydroxylated compound with potent antioxidant and anti-inflammatory activities. J. Microbiol. Biotechnol..

[10] Chang T.S., Wu J.Y., Ding H.Y., Tayo L.L., Suratos K.S., Tsai P.W., Wang T.Y., Fong Y.N., Ting H.J. (2024). Predictive production of a new highly soluble glucoside, corylin-7-*O*-*beta*-glucoside with potent anti-inflammatory and anti-melanoma activities. Appl. Biochem. Biotechnol..

[11] Chang T.S., Ding H.Y., Wang T.Y., Wu J.Y., Tsai P.W., Suratos K.S., Tayo L.L., Liu G.C., Ting H.J. (2024). In silico-guided synthesis of a new, highly soluble, and anti-melanoma flavone glucoside: Skullcapflavone II-6′-*O*-*beta*-glucoside. Biotechnol. Appl. Biochem..

[12] Ding H.Y., Wang T.Y., Wu J.Y., Zhang Y.R., Chang T.S. (2023). Novel *Ganoderma* triterpenoid saponins from the biotransformation-guided purification of a commercial *Ganoderma* extract. J. Biosci. Bioeng..

[13] Chang T.S., Ding H.Y., Wu J.Y., Wang M.L., Ting H.J. (2024). Biotransformation-guided purification of a novel glycoside derived from the extracts of Chinese berb Baizhi. J. Biosci. Bioeng..

[14] Wu J.Y., Ding H.Y., Wang T.Y., Hsu M.H., Chang T.S. (2022). A new stilbene glucoside from biotransformation-guided purification of Chinese herb Ha-Soo-Oh. Plants.

[15] Wu J.-Y., Ding H.-Y., Wang T.-Y., Cai C.-Z., Chang T.-S. (2022). Application of biotransformation-guided purification in Chinese medicine: An example to produce butin from licorice. Catalysts.

[16] Chang T.-S., Ding H.-Y., Wu J.-Y., Lee C.-C., Yang Z., Liu Y.-C., Wang T.-Y. (2025). New methyl compounds using the predicted data mining approach (PDMA), coupled with the biotransformation of *Streptomyces peucetius O*-methyltransferase. Biocatal. Biotransform..

[17] Lee S.H., Baek K., Lee J.E., Kim B.G. (2016). Using tyrosinase as a monophenol monooxygenase: A combined strategy for effective inhibition of melanin formation. Biotechnol. Bioeng..

[18] Slamova K., Kapesova J., Valentova K. (2018). “Sweet flavonoids”: Glycosidase-catalyzed modifications. Int. J. Mol. Sci..

[19] Zhao J., Yang J., Xie Y. (2019). Improvement strategies for the oral bioavailability of poorly water-soluble flavonoids: An overview. Int. J. Pharm..

[20] Wang T.H., Tseng W.C., Leu Y.L., Chen C.Y., Lee W.C., Chi Y.C., Cheng S.F., Lai C.Y., Kuo C.H., Yang S.L. (2022). The flavonoid corylin exhibits lifespan extension properties in mouse. Nat. Commun..

[21] Kopycki J.G., Rauh D., Chumanevich A.A., Neumann P., Vogt T., Stubbs M.T. (2008). Biochemical and structural analysis of substrate promiscuity in plant Mg^2+^-dependent *O*-methyltransferases. J. Mol. Biol..

[22] Kim B.G., Kim H., Hur H.G., Lim Y., Ahn J.H. (2006). Regioselectivity of 7-*O*-methyltransferase of poplar to flavones. J. Biotechnol..

[23] Panche A.N., Diwan A.D., Chandra S.R. (2016). Flavonoids: An overview. J. Nutr. Sci..

[24] Solnier J., Martin L., Bhakta S., Bucar F. (2020). Flavonoids as novel efflux pump inhibitors and antimicrobials against both environmental and pathogenic intracellular mycobacterial species. Molecules.

[25] Kim D.H., Kim B.G., Lee Y., Ryu J.Y., Lim Y., Hur H.G., Ahn J.H. (2005). Regiospecific methylation of naringenin to ponciretin by soybean *O*-methyltransferase expressed in *Escherichia coli*. J. Biotechnol..

[26] Zhang P., Zhang Z., Zhang L., Wang J., Wu C. (2020). Glycosyltransferase GT1 family: Phylogenetic distribution, substrates coverage, and representative structural features. Comput. Struct. Biotechnol. J..

[27] Tian Y., Xu W., Guang C., Zhang W., Mu W. (2023). Glycosylation of flavonoids by sucrose- and starch-utilizing glycoside hydrolases: A practical approach to enhance glycodiversification. Crit. Rev. Food Sci. Nutr..

[28] Mestrom L., Przypis M., Kowalczykiewicz D., Pollender A., Kumpf A., Marsden S.R., Bento I., Jarzebski A.B., Szymanska K., Chrusciel A. (2019). Leloir glycosyltransferases in applied biocatalysis: A multidisciplinary approach. Int. J. Mol. Sci..

[29] Lombard V., Golaconda Ramulu H., Drula E., Coutinho P.M., Henrissat B. (2014). The carbohydrate-active enzymes database (CAZy) in 2013. Nucleic Acids Res..

[30] Tian Y., Xu W., Zhang W., Zhang T., Guang C., Mu W. (2018). Amylosucrase as a transglucosylation tool: From molecular features to bioengineering applications. Biotechnol. Adv..

[31] Park H.-S., Choi K.-H., Park Y.-D., Park C.-S., Cha J.-H. (2011). Enzymatic Synthesis of Polyphenol Glycosides by Amylosucrase. J. Life Sci..

[32] Moulis C., Andre I., Remaud-Simeon M. (2016). GH13 amylosucrases and GH70 branching sucrases, atypical enzymes in their respective families. Cell. Mol. Life Sci..

[33] Rha C.S., Kim H.G., Baek N.I., Kim D.O., Park C.S. (2020). Using Amylosucrase for the Controlled Synthesis of Novel Isoquercitrin Glycosides with Different Glycosidic Linkages. J. Agric. Food Chem..

[34] Seo D.H., Yoo S.H., Choi S.J., Kim Y.R., Park C.S. (2020). Versatile biotechnological applications of amylosucrase, a novel glucosyltransferase. Food Sci. Biotechnol..

[35] Seo D.H., Jung J.H., Ha S.J., Cho H.K., Jung D.H., Kim T.J., Baek N.I., Yoo S.H., Park C.S. (2012). High-yield enzymatic bioconversion of hydroquinone to alpha-arbutin, a powerful skin lightening agent, by amylosucrase. Appl. Microbiol. Biotechnol..

[36] Cho H.K., Kim H.H., Seo D.H., Jung J.H., Park J.H., Baek N.I., Kim M.J., Yoo S.H., Cha J., Kim Y.R. (2011). Biosynthesis of (+)-catechin glycosides using recombinant amylosucrase from *Deinococcus geothermalis* DSM 11300. Enzym. Microbiol. Technol..

[37] Kim M.D., Jung D.H., Seo D.H., Jung J.H., Seo E.J., Baek N.I., Yoo S.H., Park C.S. (2016). Acceptor specificity of amylosucrase from *Deinococcus radiopugnans* and its application for synthesis of rutin derivatives. J. Microbiol. Biotechnol..

[38] Kim E.R., Rha C.S., Jung Y.S., Choi J.M., Kim G.T., Jung D.H., Kim T.J., Seo D.H., Kim D.O., Park C.S. (2019). Enzymatic modification of daidzin using heterologously expressed amylosucrase in *Bacillus subtilis*. Food Sci. Biotechnol..

[39] Rha C.S., Kim E.R., Kim Y.J., Jung Y.S., Kim D.O., Park C.S. (2019). Simple and efficient production of highly soluble daidzin glycosides by amylosucrase from *Deinococcus geothermalis*. J. Agric. Food Chem..

[40] Jung Y.S., Kim Y.J., Kim A.T., Jang D., Kim M.S., Seo D.H., Nam T.G., Rha C.S., Park C.S., Kim D.O. (2020). Enrichment of polyglucosylated isoflavones from soybean isoflavone aglycones using optimized amylosucrase transglycosylation. Molecules.

[41] Wahab S., Annadurai S., Abullais S.S., Das G., Ahmad W., Ahmad M.F., Kandasamy G., Vasudevan R., Ali M.S., Amir M. (2021). Glycyrrhiza glabra (Licorice): A comprehensive review on its phytochemistry, biological activities, clinical evidence and toxicology. Plants.

